# Precipitation forecasting utility for proactive agroecosystem management: A case study from the Texas Gulf LTAR site

**DOI:** 10.1002/jeq2.70132

**Published:** 2025-12-22

**Authors:** Merilynn C. Schantz, Stuart P. Hardegree, John T. Abatzoglou, Andrew Fullhart, Kabindra Adhikhari, R. Daren Harmel, Kelly R. Thorp, Javier Osorio Leyton, Douglas R. Smith

**Affiliations:** ^1^ USDA‐ARS Grassland Soil and Water Research Temple Texas USA; ^2^ USDA‐ARS Northwest Watershed Research Center Boise Idaho USA; ^3^ Sierra Nevada Research Institute University of California Merced California USA; ^4^ Department of Natural Resources and the Environment University of Arizona Tucson Arizona USA; ^5^ USDA‐ARS Center for Agricultural Resources Research Fort Collins Colorado USA; ^6^ Texas A&M AgriLife Research, Blackland Research and Extension Center Temple Texas USA

## Abstract

Precipitation is a primary driver of plant production across most agroecosystems. As such, enhanced precipitation forecasting will directly benefit agroecosystem management planning and decisions related to stocking rates, crop selection, restoration seedings, and wildfire fuel loads. When precipitation occurs primarily through convective storms, as is the case in the southern Great Plains, precipitation can be extremely dynamic and difficult to forecast. Providing stakeholders accurate estimates of forecast utility would provide them with the information necessary for proactive land management decision‐making. In this case study at the Texas Gulf Long‐Term Agroecosystem Research site, the objectives were to (1) optimize individual forecast models acquired from National Oceanic and Atmospheric Administration's North American Multi‐Model Ensemble and (2) assess the correlation between historical precipitation and retrospectively forecasted (hindcast) precipitation from 1982 to 2024. Optimal forecasts were often obtained from three to five aggregate combinations of individual models that were accurate for most of the year. Seasonal forecasts always produced greater forecast skill than monthly forecasts, and the best forecasts were produced in autumn and spring. Alternatively, when forecasts were acquired in autumn, forecast skill was poorer compared to when forecasts were acquired in early spring, and there were no reliable forecasts produced from 2‐month lead times at this site. El Niño Southern Oscillation conditions were, however, accurately accounted for by individual forecast models. Collectively, these findings suggest that there is utility in using precipitation forecasting for proactive agroecosystem management and planning even in dynamic precipitation regions such as the southern Great Plains.

## Core Ideas

- Forecasted monthly and seasonal precipitation can directly benefit agroecosystem management and planning.
- Quality tests are necessary to provide confidence intervals of forecast reliability to stakeholders.
- Poor forecast skill in the southern Great Plains currently limits adoption.
- Aggregating three to five individual forecast models results in improved forecast skill.
- Forecast skill of aggregated models varies by month or season, model(s), lead time, and acquisition timing.
- Aggregated model forecasts account for the ENSO events that commonly affect the region.

## Plain Language Summary

Precipitation directly affects plant production, sediment and nutrient transport, and soil health across the United States. Forecasting precipitation could benefit agroecosystem management and planning. This is especially relevant in regions with intense storm activities, like the southern Great Plains. For this study, the goal was to improve forecast skill by aggregating individual forecast models and evaluating the forecasts at a Long‐Term Agroecosystem Research site in Riesel, TX. The resulting forecasting models improved forecasts for most of the year. Forecasts were always more reliable for seasonal predictions than for monthly predictions, and the best forecasts were for autumn and spring. Forecasts were only reliable when they were acquired no more than 2 months in advance of the month or season of interest. Lastly, El Niño Southern Oscillation anomalies were accurately accounted for by individual forecast models. Collectively, these findings suggest that there is utility in using forecasting for proactive agroecosystem management.

**Abbreviations**
CanCM4i: Canadian Meteorological Center/Canadian Center for Climate Modeling and Analysis Version 4
CONUS: continental United States
ENSO: El Niño Southern Oscillation
GFDL: Geophysical Fluid Dynamics Laboratory
GFDL_SPEAR: Geophysical Fluid Dynamics Laboratory Seamless System for Prediction and Earth System Research
LTAR: Long‐Term Agroecosystem Research
MEI.v2: Multivariate ENSO Index Version 2
NMME: North American Multi‐Model Ensemble

## 1 INTRODUCTION

Accurate monthly and seasonal precipitation forecasts can directly benefit multiple agroecosystem management applications including, livestock stocking decisions, crop selection, restoration management planning, and fuel load estimations (Celis et al., 2024; Garnett & Khandekar, 2015; Klemm & McPherson, 2017; Newlands et al., 2014; Schantz et al., 2023, 2024, 2025). Adoption of precipitation forecasts into applied management planning has, however, been limited, likely due to the lack of confidence in forecast skill across time and space (Letson et al., 2001). Meredith et al. (2021), for example, suggests that land managers will likely only utilize forecasts for management and planning when (1) decision management tools are easy to access and understand, user‐friendly, and have minimal complexity; (2) there is evidence that the system provides an advantage over current management approaches; (3) the application tools have observable and testable results; (4) there is compatibility of forecasting tools with current management strategies and relevance to the localized scale of field operations; and (5) tools are adaptable and allow flexibility in decision‐making. Skill assessments, therefore, should include a confidence interval or reliability index if stakeholders are going to integrate forecasts into management applications.

To quantify and verify the utility of forecasts, correlations between historical precipitation data and precipitation forecasts (hindcasts), or herein referred to as forecasting skill, must be evaluated. Historical weather data can be obtained from a variety of sources (Parkes et al., 2019). Ground‐based and quality‐controlled weather stations provide the most reliable data for a given location (Gebremichael, 2010; Segovia‐Cardozo et al., 2023). Across scales, however, nearby ground‐based data may not be available or be representative of weather dynamics, especially in spatiotemporally complex regions, regions with intense convective storm activities, or in rural regions where the distance between individual ground‐based monitoring stations can be quite large (Essou et al., 2017; Ward et al., 2011). Gridded weather and climate data compilations have, therefore, been created using distance‐weighted average calculations among quality‐controlled ground‐based monitoring stations to provide estimated values at small, equidistant spatial scales (generally 1–4 km^2^) across the continental United States (CONUS) (Abatzoglou, 2013; Allen et al., 2021; Daly et al., 2008; Thornton et al., 2014). In previous research, Schantz et al. (in press) quantified the utility of these gridded data products for the present study site (i.e., the Texas Gulf Long‐Term Agroecosystem Research [LTAR] site in Riesel, TX) and determined that GridMET was the best gridded precipitation data source in this location as it was comprehensively complete from 1979 to 2024, and GridMET precipitation data were significantly correlated with on‐site ground‐based precipitation data. Questions remain, however, on how well individual or aggregated forecasts compare to varying historical gridded data sources or on‐site weather data; therefore, it is important to evaluate the relationship between historical data sources and forecasts to quantify forecast performance.

Monthly and seasonal precipitation forecasts can be acquired from the North American Multi‐Model Ensemble (NMME) program for 1° resolutions (∼8800 km^2^ at 45° N) across the CONUS (Kirtman et al., 2014). While these forecasts are useful in determining regional or national scale effects, coarse forecast resolutions are not generally beneficial for applied land management at field scales (E. J. Becker et al., 2022; Bolinger et al., 2017; Delworth et al., 2020). Consequently, Barbero et al. (2017) developed a downscaling procedure that allows end users to acquire forecasts for any 4 km^2^ area across the CONUS. Forecast skill is, however, often dependent on the location, time of year, month or season in which the forecast is acquired, and lead time for which the forecast is needed (Schantz et al., 2024). It is, therefore, important to evaluate forecasting skill at spatial and temporal scales relevant to local management applications (Baker et al., 2019; Barbero et al., 2017; Schantz et al., 2025). Moreover, model choice directly affects forecast skill with aggregated models commonly having better performance than individual models (Barbero et al., 2017; DelSole & Tippett, 2014; Klemm & McPherson, 2017; Ma et al., 2016; Schantz et al., 2024; Vigaud et al., 2017).

The southern Great Plains provides a unique and critical region for testing the accuracy of forecasts. This region is not only important for agricultural production, but it is also being actively urbanized and modified for renewable energy production (Londe et al., 2022). In addition, conventional agricultural management in the region makes it prone to erosion (Garbrecht et al., 2015; Hill et al., 1944). The southern Great Plains have dynamic precipitation associated with intense convective storm events, and where convective storm activities are common, precipitation forecasting skill is generally poor (Hajek & Knapp, 2022; Ojima et al., 2021). El Niño Southern Oscillation (ENSO) also has significant effects on the agroecosystems of this region (Dai & Wigley, 2000), but there are currently no clear indications of how well forecasts account for ENSO influences in regions commonly affected by ENSO conditions. These interacting and dynamic conditions make the southern Great Plains an excellent study area for utility of improved precipitation forecasts in agroecosystem management and planning; however, for wide‐scale adoption, the forecasts must be accurate enough to provide useful information at field scales.

**Core Ideas**

- Forecasted monthly and seasonal precipitation can directly benefit agroecosystem management and planning.
- Quality tests are necessary to provide confidence intervals of forecast reliability to stakeholders.
- Poor forecast skill in the southern Great Plains currently limits adoption.
- Aggregating three to five individual forecast models results in improved forecast skill.
- Forecast skill of aggregated models varies by month or season, model(s), lead time, and acquisition timing.
- Aggregated model forecasts account for the ENSO events that commonly affect the region.

In the heart of the Blackland Prairie in central Texas near the town of Riesel, an on‐site precipitation record has tracked precipitation dynamics for nearly 90 years at the historic Riesel Watersheds (Harmel et al., 2003). Data collection began in 1937 when the site was established by the US Department of Agriculture–Soil Conservation Service, now the Natural Resources Conservation Service, to analyze and understand hydrologic processes on agricultural fields and watersheds because of their impact on soil erosion, flood events, water resources, and the agricultural economy (USDA‐SCS, 1942). Given the breadth of these data, quality comparisons can be made to historical gridded products derived from calculated averages acquired from high‐end precipitation monitoring stations and forecasts associated with the NMME database, as the data used to create both the gridded and forecast models do not include this on‐farm monitoring site. Determining the utility of these forecasting products in comparison to the historical precipitation should provide critical information on spatial and temporal precipitation patterns for site‐specific management planning. Therefore, the objectives of this study were to (1) optimize individual forecast models acquired from National Oceanic and Atmospheric Administration's (NOAA) NMME by aggregating factorial combinations of individual forecasts across time and (2) assess the skill or correlation between historical on‐site or gridded precipitation data and retrospectively forecasted (hindcast) precipitation data, for each season or month, the lead time, and the acquisition month or season for all individual or aggregated hindcast models from 1982 to 2024. We hypothesized that (1) multi‐model aggregate forecasts have the greatest skill when compared to individual or ensemble (all‐combinations) forecast models; (2) optimal individual or multi‐model forecast skill varies temporally throughout months or seasons; (3) lead time does not affect forecast skill; and (4) acquisition month or season significantly affects forecast skill.

## 2 MATERIALS AND METHODS

### 2.1 Study site

**FIGURE 1:**
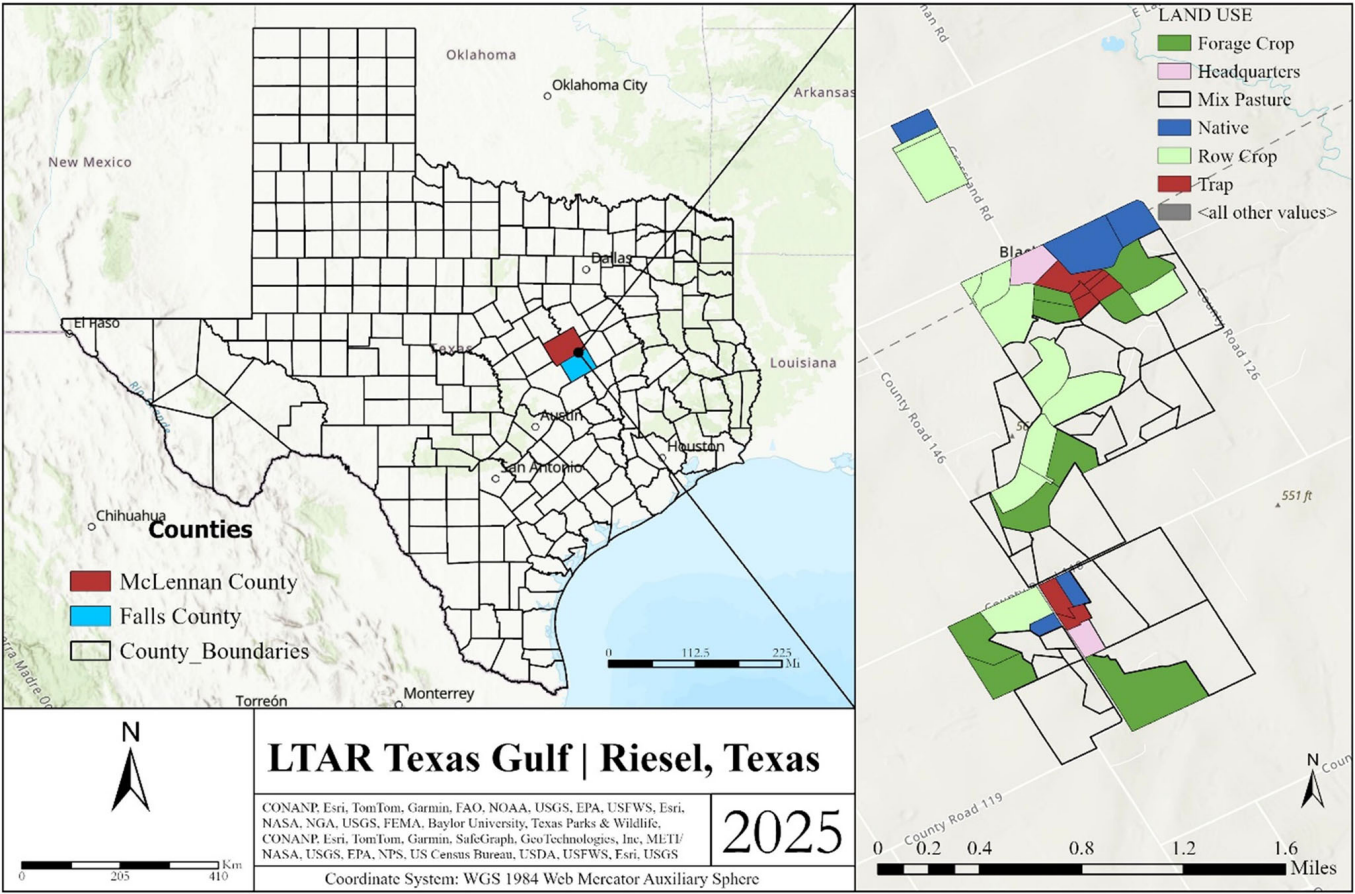
The Texas Gulf Long‐term Agroecosystem Research (LTAR) site location and associated agroecosystem land use.

This study was conducted at the Texas Gulf LTAR Network site at the US Department of Agriculture–Agricultural Research Service Grassland Soil and Water Research Laboratory watershed facility near Riesel, Texas (31.4783 N, −96.88624 W) (Figure 1). This is a key LTAR site located within the Blackland Prairies, a 4.45 million ha agricultural region extending from San Antonio 480 km north to the Red River. Across this site, Houston Black clay (very‐fine, smectitic, thermic Oxyaquic Hapluderts), noted for its strong shrink/swell potential, is the most extensive soil series. Climate in this region is characterized as continental with long, hot summers and mild winters with average annual precipitation totaling 900 mm and average daily temperatures of 25.6°C (maximum) and 13.2°C (minimum) (Figure S1). The growing season lasts on average from early March to mid‐November. Most precipitation occurs in spring and autumn, and convective thunderstorms during the summer months contribute intense, short‐duration rainfall events. Tropical hurricanes can also contribute to substantial rainfall, but their occurrence is rare. Freezing rain, sleet, and snow occur occasionally but do not contribute significant moisture. Precipitation and other hydrologic and agronomic data have been collected at the Reisel LTAR site for nearly 90 years, which permits detailed assessments of long‐term weather trends and their impacts on the local agroecosystem.

**FIGURE 2:**
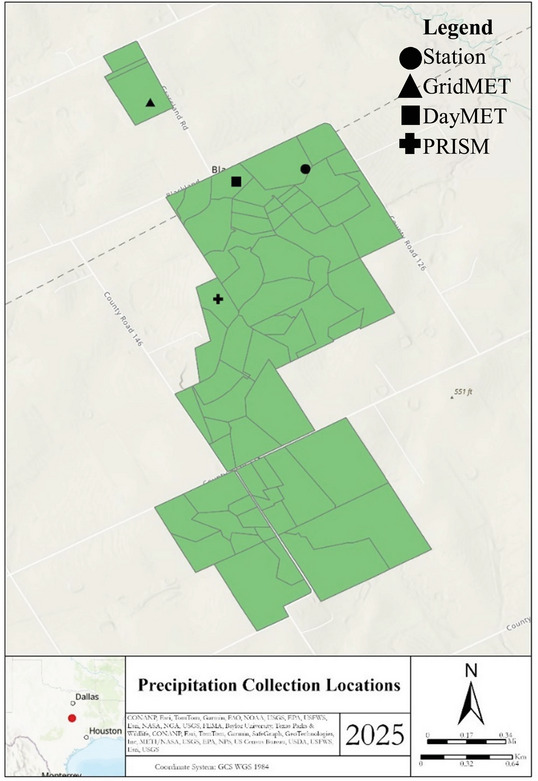
On‐site station and gridded precipitation data collection locations at the Texas Gulf Long‐Term Agroecosystem Research (LTAR) site. Points indicate the collection location of on‐site rain gauge and the centroid of gridded precipitation data acquisition sites. All forecasts were produced for the same location as the GridMET acquisition site.

Historical precipitation data were collected from a variety of sources, including gridded and on‐site weather stations. On‐site historical precipitation data used in this study were acquired from a rain gauge active from 1938 to present in a native prairie on the Riesel Watersheds (31.4791 N, −96.8819 W) (Figure 2). Gridded data were all acquired from grid points nearest to this on‐site stationary weather station. GridMET data, for example, were acquired for the location (31.483333 N, −96.891667 W) from the weather time series tool at webapps.jornada.nmsu.edu/weather (Abatzoglou, 2013). DayMET data were acquired for the location (31.4783 N, −96.88624 W) using the DayMET Single Pixel Extraction Tool at daac.ornl.gov/cgi‐bin/dsviewer.pl?ds_id=2361 (Thornton & Devarakonda, 2011). PRISM data were acquired for the location (31.4709 N, −96.8874 W) at prism.oregonstate.edu/explorer (Daly et al., 2008). In a previous study (Schantz et al., in press), no significant differences were found among these gridded data sources (Figure S2, *p *< 0.0001), but GridMET and DayMET precipitation data had stronger correlations with on‐site data.

### 2.2 Forecasts

Retrospective climate forecasts, hereafter referred to as hindcasts, were obtained from eight individual forecast models in the NMME program, as described by Kirtman et al. (2014) for 1982–2024 (Table 1). The NMME program provides experimental multi‐model seasonal forecast models on a monthly timestep for up to 12‐month lead times, which are derived from US modeling centers including NOAA/National Centers for Environmental Prediction (NCEP), NOAA/Geophysical Fluid Dynamics Laboratory (GFDL), IRI (International Research Institute), NCAR (National Center for Atmospheric Research), NASA (National Aeronautics and Space Administration), and Canada's CMC (Canadian Center for Climate) (Kirtman et al., 2014). For each of these eight individual forecast models, the average value of all factorial combinations of hindcasts was calculated, that is, (forecast model 1_2_3 = [model 1 + model 2 + model 3]/3), which resulted in 253 individual models or model combinations for each month or season across the 43 study years. For each forecast model or model combination, three factors were considered for further analysis: lead time, calendar month or season, and forecast acquisition time. Lead times of 1–9 months were first evaluated where a 1‐month lead time refers to a forecast of conditions for the following month (i.e., a 1‐month forecast acquired in January would be February). Forecast utility was also evaluated for each of the 12 calendar months and for the four seasons of the year based on climate conditions for central Texas (winter included January and February; spring included March, April, and May; summer included June, July, August, and September; and fall included October, November, and December). The utility of forecasts was determined by when they were acquired or accessed for each month or season, that is, evaluating how well a forecast acquired in January would determine February conditions.

**TABLE 1:** Forecast models supported by the North American Multimodel Ensemble as of 2025.

Model#	Code	Full model name	Citation
*1*	CFSv2	National Center for Environmental Prediction Coupled Forecast System Model Version 2	Saha et al. (2014)
*2*	GFDL	Geophysical Fluid Dynamics Laboratory CM 2.1 and 2.5	Delworth et al. (2012)
*3*	GFDL_FLOR	Geophysical Fluid Dynamics Laboratory Forecast‐Oriented Low Ocean Resolution	Vecchi et al. (2014)
*4*	NASA	National Aeronautics and Space Administration Goddard Sub‐seasonal to Seasonal Version 5	Molod et al. (2015)
*5*	NCAR	National Center for Atmospheric Research, Community Climate System Model Version 4	Kirtman et al. (personal communication)
*6*	CanCM4i	Canadian Meteorological Center/Canadian Center for Climate Modeling and Analysis Version 4	Merryfield et al. (2013)
*7*	GEM_NEMO	Global Environmental Multiscale Nucleus for European Modeling of the Ocean	Lin et al. (2020)
*8*	GFDL_SPEAR	Geophysical Fluid Dynamics Laboratory Seamless System for Prediction and Earth System Research	Delworth et al. (2020)

Precipitation hindcasts were downscaled to a 4 km^2^ resolution, and data were collected for the location (31.483333 N, −96.891667 W) following the Bias Correction‐Spatial Downscaling procedure as outlined by Barbero et al. (2017). This approach bias corrects output from each climate model's ensemble mean to the distribution of the historical range of variability (1982–2024) and spatially interpolates forecast anomalies from a 1° latitude/longitude grid of the NMME database to a 4‐km^2^ grid node.

The skill of these site‐specific forecasts was determined by the correlations (*R*) between historical on‐site and gridded climate data and retrospective forecasts (hindcasts) and their associated significance (*p*‐value) using a multivariate analysis of all factorial combinations of the eight hindcasts for all study years (1982–2024) in JMP pro (JMP Statistical Software). Monthly and seasonal correlations evaluated included the following: (1) forecast skill or the correlations between the hindcast collected for a given month or season and actual historical data acquired during that month or season, (2) lead time, or the skill of the forecast depending on how many months prior to the month of interest the forecast was made, and (3) forecast acquisition month, or season or the skill of the forecast depending on when the forecast was acquired.

We used the *R* statistic to evaluate forecast utility as it is a standard metric for assessing forecast skill and because it can be used to compare skill across large spatial and temporal scales (Barnston, 1992; E. Becker et al., 2014; Hamill & Juras, 2006). We followed the guidance of Neter et al. (1996) for estimating the statistical significance of *R* values at the *p* ≤ 0.10 level as a function of the number of years (*N*) used to develop the hindcast regression.

Gridded precipitation data were tested for their utility in forecasting along with on‐site rain gauge data for multiple reasons. (1) Historical precipitation data are rarely measured using high‐quality monitoring stations in agricultural settings, thus providing a unique opportunity to compare gridded precipitation with on‐site precipitation outputs for agricultural production and management, which we review in a corresponding paper (Schantz et al., in press), (2) Evaluating both gridded and on‐site precipitation provides detailed information on whether there are important differences in forecast skill when verified by differing precipitation datasets, and (3) Precipitation is spatiotemporally variable in the Southern Great Plains and especially at this site; thus, validating the utility of forecasts using these multiple historical datasets provides credence to incorporating forecasts into agricultural management planning.

### 2.3 El Niño Southern Oscillation

ENSO effects on forecast utility were also evaluated in this study by first obtaining ENSO data from the Multivariate ENSO Index Version 2 (MEI.v2) rank at https://psl.noaa.gov/enso/mei (Wolter & Timlin, 1998; Zhang et al., 2019). The multivariate index is calculated using six variables, including sea‐level pressure, zonal and meridional components of the surface wind, sea surface temperature, surface air temperature, and total cloudiness fraction of the sky (Wolter & Timlin, 1993). The MEI.v2 expands upon this initial assessment by calculating a time series of conditions over the tropical Pacific Basin using five of these variables, including sea level pressure, sea surface temperature, surface zonal winds, surface meridional winds, and outgoing longwave radiation, to produce a bimonthly time series of ENSO conditions from 1979 to present and reduce interseason variability (Kobayashi et al., 2015; Zhang et al., 2019). These data include a quantitative value on the intensity of an El Niño or La Niña event, which ranges from −3 to 3 where −3 refers to a very strong La Niña event, while 3 refers to a very strong El Niño event (Wolter & Timlin, 1998; Zhang et al., 2019). For this analysis, a regression model compared the monthly historical precipitation to the published ENSO MEI.v2 rank. A second regression model was used to compare the eight independent forecast models (Table 1) to the MEI.v2 to determine if forecasts were accounting for ENSO events.

## 3 RESULTS

### 3.1 Forecasts

#### 3.1.1 Forecast skill

Monthly skill assessments indicated that all months, except for August, had at least one forecast model or model combination with significant correlations with historical precipitation data (Table 2). Aggregate combinations of three to five models were often the top performers for the month, but there were a few cases where individual models had greater forecast skill than aggregated combinations. There were no cases where the full aggregated combination, or ensemble mean, was the best performing forecast across historical data sources. While the best performing forecast model or model combination varied by historical data source, there were several months where the top performing forecast model was the same across historical data sources (e.g., January, May, June, and October). It was also common for similar individual models to populate the best performing aggregate model combination across data sources. For example, in February, the top performer for GridMET, PRISM, and on‐site station data was the aggregated model 1_2_6_7_8, while the top performer for DayMET was model combination 2_6_7_8 (see Table 1 for forecast names). Forecasting skill varied throughout the year but was greatest in July where *R* averaged 0.6306, and in December with an average *R* of 0.6191 (*p *< 0.05). Alternatively, forecast skill was least in August where no models were significant, and *R* averaged only 0.0857. There were few differences among historical data sources, but the average forecast skill across the year was greatest for both GridMET and DayMET (*R* = 0.4638), followed by PRISM (*R* = 0.4498) and on‐site station data (*R* = 0.4370) (*p *< 0.05).

**TABLE 2:** Monthly skill assessments. This table presents the hindcast model(s) with the best skill, or correlation between historical data and hindcasts, at 2‐month lead times. Hindcast models are either individual where the model number is referred to in parentheses following the model name, or they are an aggregate combination of the eight individual hindcast models as indicated by the number combinations (see Table 1 for forecast names). Historical data are either from gridded databases or on‐site station data.

	GridMET			DayMET		
	Best model(s)	*R*	*p*‐value	Best model(s)	*R*	*p*‐value
January	1_2_6_8	**0.4019**	**0.0083**	1_2_6_8	**0.4461**	**0.0031**
February	1_2_6_7_8	**0.5595**	**0.0001**	2_6_7_8	**0.5125**	**0.0004**
March	CFSv2 (1)	**0.4039**	**0.0001**	1_6	**0.3684**	**0.0008**
April	5_6_7	**0.4713**	**<0.0001**	5_6_7	**0.4345**	**<0.0001**
May	3_6_7_8	**0.5102**	**<0.0001**	3_6_7_8	**0.4996**	**<0.0001**
June	NASA (4)	**0.4191**	**<0.0001**	NASA (4)	**0.3600**	**0.0008**
July	1_2_4_5_6_8	**0.6257**	**<0.0001**	1_2_3_4_5_8	**0.6150**	**<0.0001**
August	2_6	0.0960	0.4657	CFSv2 (1)	0.0880	0.4261
September	2_4_8	**0.3754**	**0.0143**	GFDL_SPEAR (8)	**0.3670**	**0.0024**
October	2_5_8	**0.5277**	**0.0003**	2_5_8	**0.4954**	**0.0009**
November	3_4_6_8	**0.5580**	**<0.0001**	2_3_4_6_8	**0.5507**	**0.0002**
December	1_2_3_4_6_7_8	**0.6168**	**<0.0001**	1_2_3_4_6_7_8	**0.6121**	**<0.0001**
	PRISM			Station		
	Best model(s)	*R*	*p*‐value	Best model(s)	*R*	*p*‐value
January	1_2_6_8	**0.3542**	**0.0214**	1_2_6_8	**0.4690**	**0.0017**
February	1_2_6_7_8	**0.5027**	**0.0007**	1_2_6_7_8	**0.5114**	**0.0005**
March	1_6	**0.3137**	**0.0046**	1_6	**0.3586**	**0.0015**
April	3_5_6_7_8	**0.5176**	**<0.0001**	3_8	**0.5211**	**<0.0001**
May	3_6_7_8	**0.4410**	**0.0004**	3_6_7_8	**0.5121**	**<.0001**
June	NASA (4)	**0.3910**	**0.0002**	NASA (4)	**0.3095**	**0.0065**
July	1_2_4_5_6_8	**0.6600**	**<.0001**	1_2_4_6_8	**0.6218**	**<.0001**
August	2_3_6	0.1418	0.2798	1_2_3	0.0169	0.8979
September	GFDL_SPEAR (8)	**0.3745**	**0.0017**	1_2_4_8	**0.3191**	**0.0394**
October	2_5_8	**0.5096**	**0.0006**	2_5_8	**0.4692**	**0.0017**
November	3_4_6_8	**0.5559**	**<0.0001**	3_4_6_8	**0.5228**	**<0.0001**
December	1_2_3_4_6_7_8	**0.6357**	**<0.0001**	1_2_3_4_7_8	**0.6119**	**<0.0001**

*Note*: Bold values indicate significant correlations (*p* < 0.05).

Abbreviations: CFSv2, National Center for Environmental Prediction Coupled Forecast System Model Version 2; GFDL_SPEAR, Geophysical Fluid Dynamics Laboratory Seamless System for Prediction and Earth System Research; NASA, National Aeronautics and Space Administration Goddard Sub‐seasonal to Seasonal Version 5.

Seasonal skill assessments indicated that while there were many significant individual or ensemble mean forecasts, the best performance often occurred with aggregate combinations of four to five individual forecast models (Table 3). While the best performing forecast model combination varied throughout the year, the individual model Geophysical Fluid Dynamics Laboratory Seamless System for Prediction and Earth System Research (GFDL_SPEAR) (8) was always present in each aggregation of the best performing model(s) at this site. When ranked by the total number of forecast models, individual models evaluated by season were the 95–225 best models out of the 253 total models evaluated, while the ensemble mean forecasts ranked 80–211 best models out of the 253 total models evaluated by season. Across all forecast models, the greatest correlations between historical and forecasted data occurred in autumn (average *R* = 0.7192), followed by spring (average *R* = 0.6155), and performed similarly well in both winter and summer (average *R* = 0.5305, 0.5136) (*p *< 0.05). Forecast skill only varied slightly by historical data choice where GridMET data had, on average, the greatest correlation with forecasts (*R* = 0.6164), followed by DayMET (*R* = 0.5982), station data (*R* = 0.5835), and PRISM (*R* = 0.5809) (*p *< 0.05).

**TABLE 3:** Seasonal skill assessments. This table presents the hindcast model(s) skill (*R*), or correlation between historical data and hindcasts, with the highest correlation value and the associated significance test (*p*‐values) at 2‐month lead times. Hindcast models are either individual where the model number is referred to in parentheses following the model's name, or they are an aggregate combination of the eight individual hindcast models as indicated by the number combinations (see Table 1 for forecast information). Historical data are from gridded databases, GridMET, DayMET, or PRISM, or from on‐site station data, that is, Station. Rank indicates how good the model(s) were in comparison to the potential 253 model(s) evaluated. Subpart A indicates the best‐fitting model(s), subpart B indicates the best individual model, and subpart C indicates all model combinations, or the ensemble mean.

	*GridMET*			*DayMET*		
*(A)*	Best model(s)	*R*	*p*‐value	Best model(s)	*R*	*p*‐value
*Fall*	2_4_7_8	**0.7350**	**<0.0001**	2_4_8	**0.7329**	**<0.0001**
*Spring*	1_5_6_7_8	**0.6438**	**<0.0001**	1_3_5_6_7_8	**0.5837**	**<0.0001**
*Summer*	2_4_5_6_8	**0.5428**	**0.0002**	2_4_5_8	**0.5203**	**0.0004**
*Winter*	1_2_6_8	**0.5440**	**0.0002**	1_2_6_8	**0.5557**	**0.0001**
	PRISM			Station		
	Best model(s)	*R*	*p*‐value	Best model(s)	*R*	*p*‐value
*Fall*	2_4_7_8	**0.7229**	**<0.0001**	2_4_8	**0.6861**	**<0.0001**
*Spring*	3_5_6_8	**0.6053**	**<0.0001**	3_5_6_7_8	**0.6293**	**<0.0001**
*Summer*	2_4_5_8	**0.5020**	**0.0007**	2_4_5_6_8	**0.4894**	**0.0010**
*Winter*	1_2_6_8	**0.4933**	**0.0009**	1_2_6_8	**0.5290**	**0.0003**

		Rank/253 models								
*(B)*	Best individual model		GridMET		DayMET		PRISM		Station	
*Fall*	GFDL (2)	225	**0.5385**	**<0.0001**	**0.5051**	**<0.0001**	**0.5144**	**<0.0001**	**0.4458**	**0.0004**
*Spring*	GEM_NEMO (7)	100	**0.5007**	**<0.0001**	**0.4209**	**0.0001**	**0.3910**	**0.0003**	**0.5143**	**<0.0001**
*Summer*	CanCM4i (6)	95	**0.3991**	**0.0002**	**0.3263**	**0.0031**	**0.3096**	**0.0052**	**0.2762**	**0.0157**
*Winter*	GFDL (2)	153	**0.2695**	**0.0342**	0.2484	0.0515	0.2017	0.1160	**0.2752**	**0.0304**
**(** *C* **)**	All models									
*Fall*	1_2_3_4_5_6_7_8	165	**0.5866**	**<0.0001**	**0.5719**	**<0.0001**	**0.5741**	**<0.0001**	**0.5228**	**<0.0001**
*Spring*	1_2_3_4_5_6_7_8	80	**0.5340**	**<0.0001**	**0.4754**	**<0.0001**	**0.4697**	**<0.0001**	**0.5369**	**<0.0001**
*Summer*	1_2_3_4_5_6_7_8	161	**0.3382**	**0.0014**	**0.3029**	**0.0051**	**0.2832**	**0.0082**	**0.2937**	**0.0100**
Winter	1_2_3_4_5_6_7_8	211	0.2091	0.0533	0.1781	0.1050	0.1747	0.1077	**0.2455**	**0.0326**

*Note*: Bold values indicate significant correlations (*p* < 0.05).

Abbreviations: CanCM4i, Canadian Meteorological Center/Canadian Center for Climate Modeling and Analysis Version 4; GEM_NEMO, Global Environmental Multiscale Nucleus for European Modeling of the Ocean; GFDL, Geophysical Fluid Dynamics Laboratory.

#### 3.1.2 Lead time

The average of all correlations between historical gridded or stationary precipitation data and hindcasts for monthly precipitation analyses was only significant 1 month in advance (lead time = 1 month) of the forecast month of interest (Table 4A; *p *< 0.05). Correlations between historical gridded or stationary precipitation data and hindcasts at 2‐month lead times were significant at the 90% interval (*p *< 0.10) when hindcasts were compared to the GridMET and station data and were significant at the 95% level (*p *< 0.05) when hindcasts were compared to DayMET data (Table 4). There were no significant correlations among hindcasts and historical weather data for any other lead time for monthly precipitation.

**TABLE 4:** Average correlations between historical gridded or on‐site weather station precipitation data and hindcasts across monthly (subpart A) and seasonal (subpart B) analyses sorted by the lead time (1–9 months in advance of month of interest).

(A)	*GridMET*		*DayMET*		*PRISM*		*Station*	
*Monthly hindcasts*	*R*	*p*‐value	*R*	*p*‐value	*R*	*p*‐value	*R*	*p*‐value
Lead 1	**0.3170**	**0.0003**	**0.3038**	**0.0003**	**0.2816**	**0.0022**	**0.2706**	**0.0012**
Lead 2	0.1175	0.0530	**0.1249**	**0.0340**	0.0803	0.1882	0.1083	0.0716
Lead 3	−0.0131	0.6399	0.0054	0.6386	−0.0283	0.6041	−0.0253	0.5949
Lead 4	−0.0808	0.2222	−0.0739	0.2773	−0.0809	0.1838	−0.0764	0.2315
Lead 5	−0.0481	0.4217	−0.0435	0.4395	−0.0451	0.4352	−0.0398	0.4753
Lead 6	0.0525	0.3826	0.0460	0.4266	0.0524	0.3631	0.0446	0.4335
Lead 7	0.1119	0.0564	0.0956	0.1168	0.1067	0.0754	0.0953	0.1287
Lead 8	0.0108	0.6680	−0.0118	0.5656	0.0164	0.6851	0.0017	0.6263
Lead 9	−0.0692	0.2321	−0.0915	0.1231	−0.0442	0.4324	−0.0350	0.5108

*(B)*	GridMET		DayMET		PRISM		Station	
*Seasonal hindcasts*	*R*	*p*‐value	*R*	*p*‐value	*R*	*p*‐value	*R*	*p*‐value
*Lead 1*	**0.5024**	**0.0003**	**0.5027**	**0.0003**	**0.4539**	**0.0009**	**0.4783**	**0.0003**
*Lead 2*	**0.2524**	**0.0123**	**0.2821**	**0.0038**	**0.2099**	**0.0397**	**0.2633**	**0.0071**
*Lead 3*	0.0148	0.7056	0.0656	0.5162	−0.0024	0.7298	0.0442	0.6446
*Lead 4*	−0.0883	0.4209	−0.0542	0.4831	−0.0983	0.3734	−0.0607	0.5406
*Lead 5*	−0.0066	0.5728	0.0102	0.5509	−0.0075	0.6585	−0.0018	0.7476
*Lead 6*	0.1176	0.2861	0.0987	0.3557	0.1239	0.2323	0.1056	0.2849
*Lead 7*	0.1732	0.0906	0.1364	0.1750	0.1775	0.0816	0.1588	0.1160
*Lead 8*	0.0684	0.4802	0.0222	0.7193	0.0850	0.3788	0.0695	0.4913
*Lead 9*	−0.0374	0.5920	−0.0751	0.4405	0.0043	0.7250	−0.0008	0.6878

*Note*: Bold values indicate significant correlations (*p* < 0.05).

Seasonal precipitation analyses identified that average correlations between historical gridded or stationary precipitation data and hindcasts were significant across all gridded or station data for the first two lead times (1 or 2 months in advance of the forecast month) (Table 4B; *p *< 0.05). There were no significant correlations beyond 2‐month lead times in the seasonal analyses.

#### 3.1.3 Acquisition time

Acquiring forecasts in specific months or seasons can directly affect forecast skill in that a forecast is only reliable when average correlations between historical and forecasted precipitation data for the acquisition month or season have a high reliability index, which was set as >90% in this study (Table 5; *p *< 0.10). Monthly forecast acquisition skill appeared to be cyclical since the average correlations of all models for a given month were only significant for January, April, May, August, October, November, and for some historical datasets in December (*p *< 0.10). Acquisition time was more reliable for seasonal models, where acquiring forecasts in fall, spring, or summer always produced a reliable forecast, but acquiring forecasts in winter was only useful when historical on‐site station data were correlated with forecast models.

**TABLE 5:** Average monthly (subpart A) and seasonal (subpart B) hindcast skill (*R*) and associated significant tests (*p*‐values) by acquisition time, or time in which the forecast is acquired at 2‐month lead intervals for each gridded (i.e., GridMET, DayMET, and PRISM) or on‐site weather station data (i.e., station and dataset).

(A)	GridMET		DayMET		PRISM		Station	
Monthly hindcasts	*R*	*p*‐value	*R*	*p*‐value	*R*	*p*‐value	*R*	*p*‐value
Jan	**0.3993**	**0.0040**	**0.3224**	**0.0202**	**0.3572**	**0.0094**	**0.4097**	**0.0046**
Feb	0.1959	0.2601	0.1551	0.3328	0.1694	0.3039	0.2133	0.2250
Mar	−0.0744	0.4460	−0.0963	0.4352	−0.1084	0.4011	−0.1119	0.4035
Apr	**0.3449**	**0.0724**	**0.3183**	**0.0890**	**0.2756**	**0.1255**	**0.3066**	**0.0686**
May	**0.3606**	**0.0188**	**0.3252**	**0.0341**	**0.3478**	**0.0260**	**0.2623**	**0.0764**
Jun	0.2110	0.1770	0.2088	0.1771	0.2486	0.0994	0.1880	0.2117
Jul	0.0367	0.6379	0.0438	0.6345	0.1032	0.4531	−0.0333	0.7701
Aug	**0.3761**	**0.0093**	**0.3614**	**0.0119**	**0.3204**	**0.0216**	**0.3734**	**0.0184**
Sept	0.1958	0.2016	0.1734	0.2542	0.1910	0.2289	0.1367	0.3713
Oct	**0.4957**	**0.0028**	**0.4807**	**0.0027**	**0.4705**	**0.0035**	**0.4855**	**0.0024**
Nov	**0.3088**	**0.0667**	**0.3093**	**0.0582**	**0.3109**	**0.0666**	**0.3078**	**0.0529**
Dec	0.2288	0.1043	**0.2399**	**0.0981**	0.2155	0.1320	**0.2948**	**0.0404**

(B)	GridMET		DayMET		PRISM		Station	
Seasonal hindcasts	*R*	*p*‐value	*R*	*p*‐value	*R*	*p*‐value	*R*	*p*‐value
Fall	**0.3002**	**0.0524**	**0.2961**	**0.0661**	**0.2733**	**0.0744**	**0.3279**	**0.0345**
Spring	**0.3541**	**0.0364**	**0.3158**	**0.0540**	**0.3125**	**0.0600**	**0.3164**	**0.0505**
Summer	**0.6072**	**0.0003**	**0.5960**	**0.0013**	**0.5870**	**0.0006**	**0.5394**	**0.0005**
Winter	0.3771	0.1654	0.3172	0.2890	0.3297	0.2112	**0.4251**	**0.0487**

*Note*: Bold values indicate significant correlations (*p* < 0.10).

### 3.2 El Niño Southern Oscillation

**FIGURE 3:**
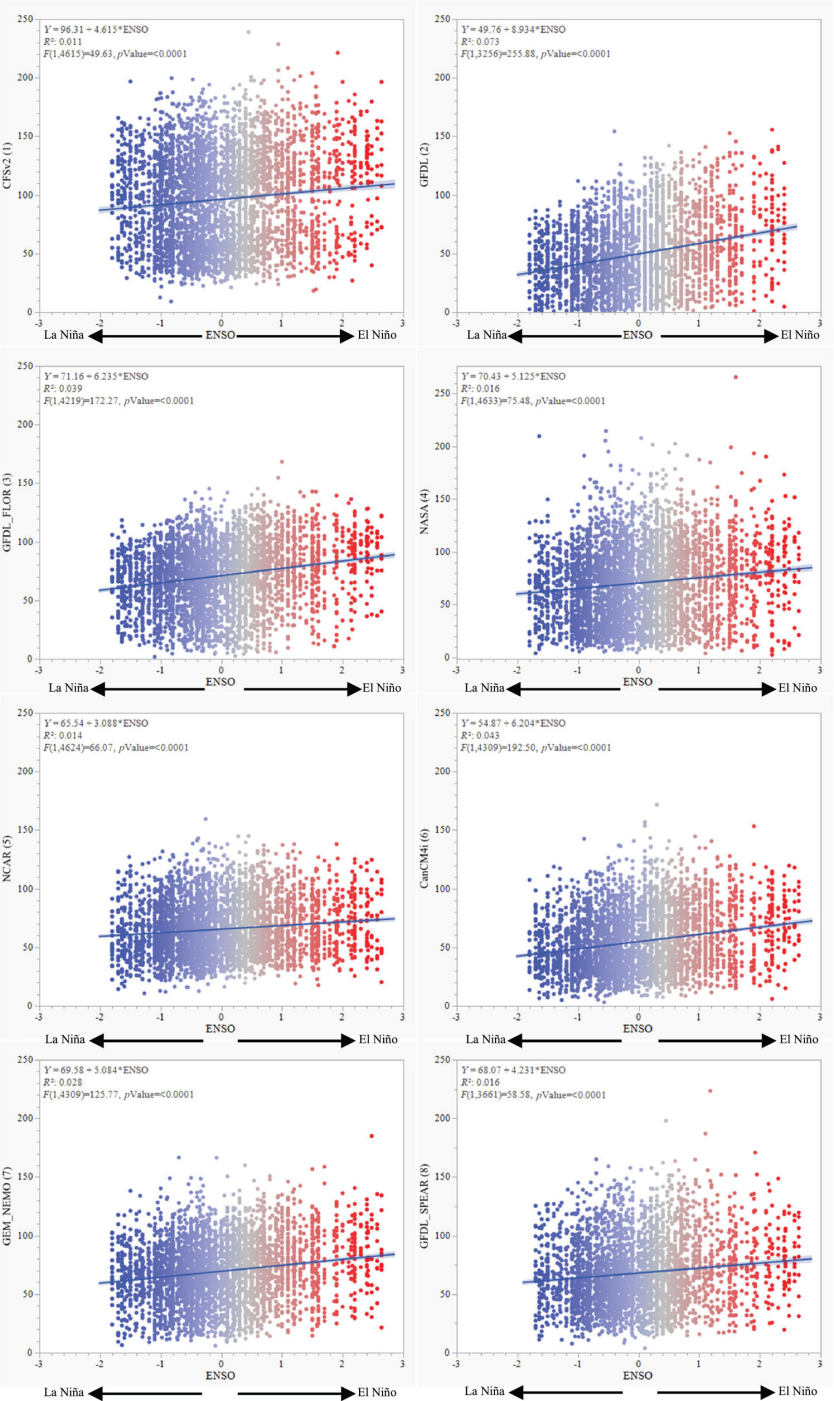
Forecasted monthly precipitation (mm) for the Texas Gulf Long‐Term Agroecosystem Research (LTAR) site in Riesel, TX, by El Niño Southern Oscillation (ENSO) Multivariate ENSO Index Version 2 (MEI.v2) rank. Individual figures represent the eight individual monthly forecast model outputs (see Table 1 for forecast model information). Colors indicate the ENSO MEI.v2 rank from very severe El Niño in red to severe La Niña in blue. All models were significant at *p *< 0.0001.

There were strong relationships between forecasted precipitation and ENSO events. Individual forecasts models, for example, all showed a positive and significant relationship between ENSO conditions and precipitation forecasts (*p *< 0.0001) and regressions between individual forecast models and ENSO indicated that the Canadian Meteorological Center/Canadian Center for Climate Modeling and Analysis Version 4 (CanCM4i) had the greatest *R* value of 0.043 while the poorest model was the National Center for Environmental Prediction Coupled Forecast System Model Version 2 (CFSv2) with an *R* of only 0.011 (Figure 3).

## 4 DISCUSSION

Forecasting utility for site or region‐specific management applications can be evaluated by utilizing statistically downscaled monthly or seasonal weather forecasts (Barbero et al., 2017; Schantz et al., 2024). Since multiple monthly or seasonal forecast models exist, not knowing which one(s) are best limits their utilization as an agricultural management decision support tool (Klemm & McPherson, 2017). In support of our first hypothesis and multiple previous studies, aggregating forecast models often had greater skill compared to individual forecast models (Tables 2 and 3). While others have found synergistic skill in forecast aggregation, primarily through an all‐model “ensemble” combination (Klemm & McPherson, 2017; Vigaud et al., 2017), the ensemble model in the present study was never the optimal model choice for either monthly or seasonal evaluations. Instead, the optimal forecast was most often an aggregate combination of four to five individual models, which was the same conclusion reached by Schantz et al. (2024).

At this study site, we identified that one forecast (i.e., Model 8, the GFDL_SPEAR Model, Tables 2 and 3), performed well across months and seasons. Interestingly, even though this model was frequently in the optimal model aggregation, it was never the best individual model across seasons (Table 3). Other models that continued to appear as the optimal individual or aggregate forecast combination across months or seasons for this area included Models 2 (GFDL), 7 (Global Environmental Multiscale Nucleus for European Modeling of the Ocean [GEM_NEMO]), 6 (CanCM4i), and 4 (NASA). Individual forecasts tend to be optimized for a specific region or time of interest and often for the region or application for which they were created (Klemm & McPherson, 2017). Multi‐model aggregations, alternatively, have been noted as resulting in synergistic skill (i.e., Schantz et al., 2024). Consequently, while individual models can produce a quality forecast for site specific management applications, the identification of top performing individual forecasts that can be melded into forecast aggregations can improve forecasting skill and thus the reliability of forecasting for agricultural management planning.

For individual locations, historical precipitation data can be obtained from an on‐site weather station or from gridded weather and climate databases (e.g., GridMET, DayMET, and PRISM) (Abatzoglou, 2013; Daly et al., 2008; Thornton et al., 2014); however, there can be some differences between the measured and gridded data, such as lower precipitation in PRISM compared to GridMET, DayMET, and measured on‐site data (Schantz et al., in press). Results of the present study, however, showed that the optimal model choice was similar among historical gridded or station datasets (Figure 3), likely because the data from the historical weather sources were significantly correlated (Figure S1). Consequently, while the optimal model or model combination may differ slightly over time, there were several individual or aggregated models that were optimized for the site and/or time of interest that could be relied upon to provide quality and reliable forecasts.

Subscribers to an NOAA prediction tool suggest that lead time is the most important variable distinguishing those who use the prediction in decision‐making from those who do not (Barnes et al., 2023; Ghamariadyn & Imteaz, 2021; Easterling & Mjelde, 1987). The lack of accuracy across lead times is also a major deterrent to the use of forecasts and adoption by agricultural producers (Barnston et al., 1996; Nyamekye et al., 2021; Westra & Sarma, 2010). Contrary to our third hypothesis that lead time would not affect forecast skill, monthly or seasonal forecasts were only accurate at up to 2‐month lead times suggesting that management decisions made for this region should occur throughout the year but only 2 months in advance. This result is especially important when producers make critical management decisions, such as setting stocking rates and seed purchasing in late winter or early spring. Enhanced knowledge is especially important for non‐irrigated producers who rely on natural precipitation to support crop or forage production, as these producers are the most likely users of forecasting tools, and the lead time information could add significant value to their operations (Nyamekye et al., 2021).

Forecast acquisition timing can considerably affect forecast accuracy (Schantz et al., 2023), and both Barbero et al. (2017) and Schantz et al. (2024) suggested that ENSO conditions can affect forecast skill by skewing values during winters in which ENSO conditions are strong. Similarly, this study suggests that poorer forecast skill of forecasts acquired in winter months, when compared to other seasons, is likely due to ENSO anomalies and their associated effects on sea level pressure, sea surface temperature, surface zonal winds, surface meridional winds, and outgoing longwave radiation (Delworth et al., 2020, 2012; Lin et al., 2020; Merryfield et al., 2013; Molod et al., 2015; Saha et al., 2014; Vecchi et al., 2014).

Quantifying the effects of ENSO conditions on forecast skill is necessary to determine the times and places where forecasts are good enough to integrate into management planning (Hardegree et al., 2018; Meredith et al., 2021). In the southern Great Plains, El Niño conditions and their effects commonly increase precipitation by approximately 50 mm compared to normal years, and precipitation increases during El Niño commonly occur during the winter season (Dai & Wigley, 2000). Lepore et al. (2017) and Goddard and Gershunov et al. (2020) also indicate that ENSO events are increasing and suggest that ENSO events cause extreme weather and climate events around the globe. In this study, each of the individual models associated with the NMME database accurately accounted for ENSO conditions (Figure 3). Consequently, even if ENSO conditions increase, forecast models should still provide reliable enough estimates to be useful for agroecosystem management planning, decision tools, and models.

Some caveats to consider when interpreting our results or choosing to incorporate forecasts into management planning are as follows: (1) Forecasting is an imprecise science and will vary spatiotemporally. Forecast assessments performed at coarse spatial or temporal ranges can, for example, produce irrelevant results for site‐ or time‐specific applications (Feldmann et al., 2019; Liu et al., 2022; Roy et al., 2020). While the evaluations at this site determined multiple individual or aggregate models that performed well across specific months or season, there were times that continued to produce poor performing forecasts, likely due to variable spatiotemporal climate anomalies. Spring precipitation in the Southern Great Plains, for example, can be highly variable from year‐to‐year and late summer hurricanes also contribute to variable precipitation in this region (Harmel et al., 2003). While many spring forecasts were found to be useful in this study (Tables 2 and 3), summer, and particularly August, precipitation forecasts were poor, likely due to hurricane activity and their associated effects on precipitation in this region. (2) Specific months or seasons may produce high forecast skill but not be relevant for management planning activities. We identified that both July and December had high forecast skill, likely due to low historical precipitation occurrences in these months (Figure 2 and Figure S1). While this information can be interesting, the number of agricultural management activities that commonly occur in July and/or December in the Southern Great Plains is low and may not be as relevant for management planning as spring precipitation events. (3) Forecasting precipitation should be done at 1‐ or 2‐month lead times for sites like the Southern Great Plains as long lead times can yield inaccurate results (Barbero et al., 2017; Schantz et al., 2024). For this study we limited our forecasting skill assessments to 2‐month lead times (Tables 2 and 3) as forecasts acquired for more than 2‐month lead times were not found to be accurate.

## 5 CONCLUSION

Seasonal climate variability has enormous impacts on agricultural productivity, rural livelihoods, and economics at farm, regional, and national scales given that farmers are frequently forced to make adaptive management decisions in the face of this variability. Minimizing crop and forage production losses in droughts and deluge conditions while taking advantage of favorable seasons is the promise of seasonal climate forecasts; however, seasonal forecasts must first be shown to be reliable before producers will integrate them into their management and planning (Meredith et al., 2021).

Site specific forecast evaluations are important for determining forecast reliability across spatiotemporal scales for estimating end‐of‐year forage or crop production (Schantz et al., 2025; Smith et al., 2000). The results of this research indicate that forecasts could be reliably produced across most months and seasons even in a dynamic climate region that experiences high inter‐annual rainfall variability and is strongly influenced by ENSO events. Forecasts, therefore, have considerable potential to positively inform agricultural decisions and agricultural models. The finding that forecasts are only reliable at 1–2‐month lead times and only when acquired during fall, spring, or summer highlights the importance of understanding their limitation when integrating forecasts into management planning.

## AUTHOR CONTRIBUTIONS

**Merilynn C. Schantz**: Conceptualization; formal analysis; writing—original draft. **Stuart P. Hardegree**: Conceptualization; methodology. **John T. Abatzoglou**: Resources; software. **Andrew Fullhart**: Writing—review and editing. **Kabindra Adhikhari**: Formal analysis. **R. Daren Harmel**: Writing—review and editing. **Kelly R. Thorp**: Writing—review and editing. **Javier Osorio Leyton**: Resources. **Douglas R. Smith**: Writing—review and editing.

## CONFLICT OF INTEREST STATEMENT

The authors declare no conflicts of interest.

## Supporting information




**Supplementary Figure 1**. Historical mean (± standard error) monthly precipitation (mm) (Figure A, B) and temperature (Figure C, D) for all weather data sources. Figure A refers to the total monthly precipitation and Figure B figures refers to the year (1982‐2024). Figure C refers to the average station monthly temperatures and Figure D to the average station yearly temperatures.
**Supplementary Figure 2**. Regressions between daily on‐site weather station and three gridded weather datasets; GridMET, DayMET, and PRISM. Figure A indicates the regression between historical weather data sources, i.e., on‐site weather station precipitation, i.e.,‘Station’ compared to the gridded products, GridMET, DayMET and PRISM. Figure B refers to the regression between the residual error value of each gridded data source where residual error = gridded‐station precipitation data when compared to the on‐site weather station precipitation (mm‐month^−1^). For both figures, lines represent the regression and the surrounding like colors represent the confidence interval. Regressions were significantly different when *p *< 0.05.
