# Supplementary material for: Precipitation forecasting utility for proactive agroecosystem management: A case study from the Texas Gulf LTAR site
Source: J Environ Qual. 2025 Dec 22;55(1):e70132. doi: 10.1002/jeq2.70132 (PMC12723340; doi:10.1002/jeq2.70132)
Supplement: Supplementary file 1 — Supplementary Figure 1. Historical mean (± standard error) monthly precipitation (mm) (Figure A, B) and temperature (Figure C, D) for all weather data sources. Figure A refers to the total monthly precipitation and Figure B figures refers to the year (1982‐2024). Figure C refers to the average station monthly temperatures and Figure D to the average station yearly temperatures. Supplementary Figure 2. Regressions between daily on‐site weather station and three gridded weather datasets; GridMET, DayMET, and PRISM. Figure A indicates the regression between historical weather data sources, i.e., on‐site weather station precipitation, i.e.,‘Station’ compared to the gridded products, GridMET, DayMET and PRISM. Figure B refers to the regression between the residual error value of each gridded data source where residual error = gridded‐station precipitation data when compared to the on‐site weather station precipitation (mm‐month−1). For both figures, lines represent the regression and the surrounding like colors represent the confidence interval. Regressions were significantly different when p < 0.05. [file JEQ2-55-0-s001.docx]

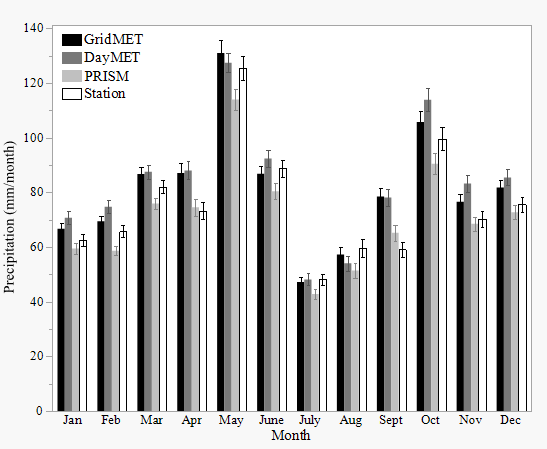

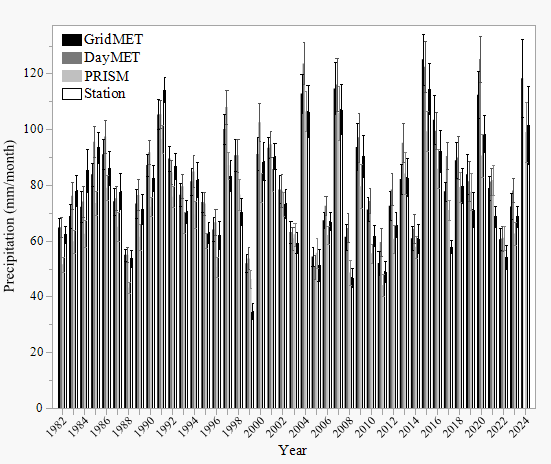


B

A


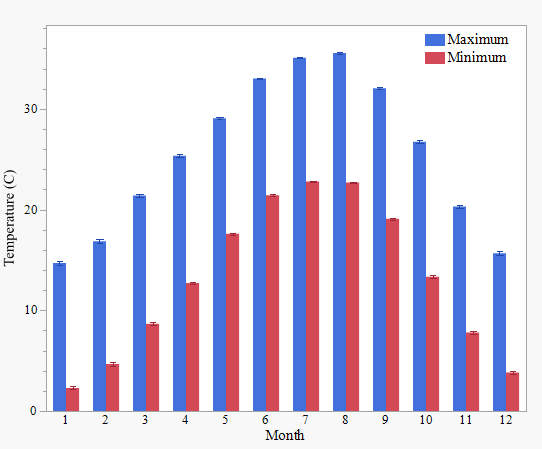

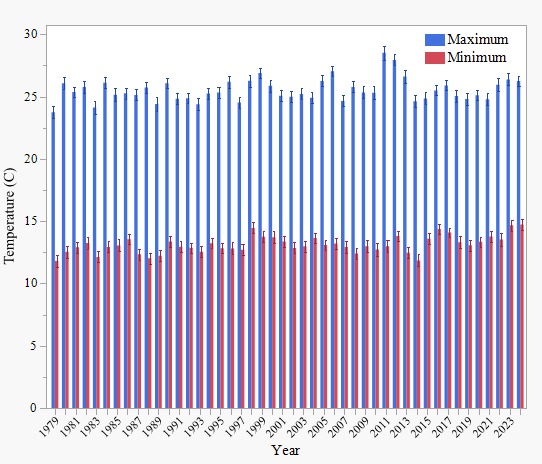


C

D

**Supplementary Figure 1.** Historical mean (± standard error) monthly precipitation (mm) (Figure A, B) and temperature (Figure C, D) for all weather data sources. Figure A refers to the total monthly precipitation and Figure B figures refers to the year (1982-2024). Figure C refers to the average station monthly temperatures and Figure D to the average station yearly temperatures.

B4


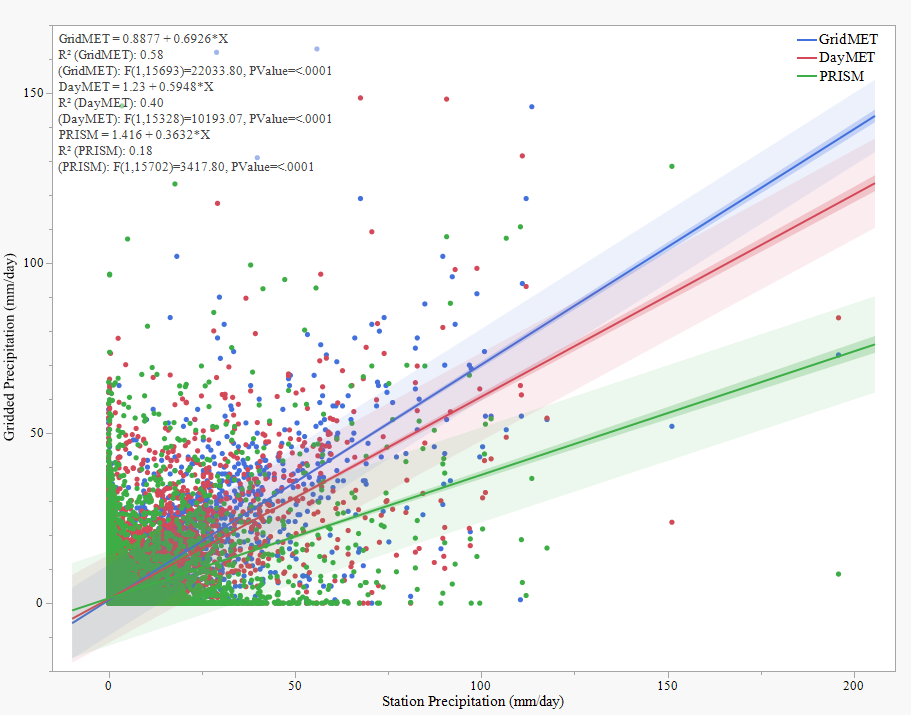

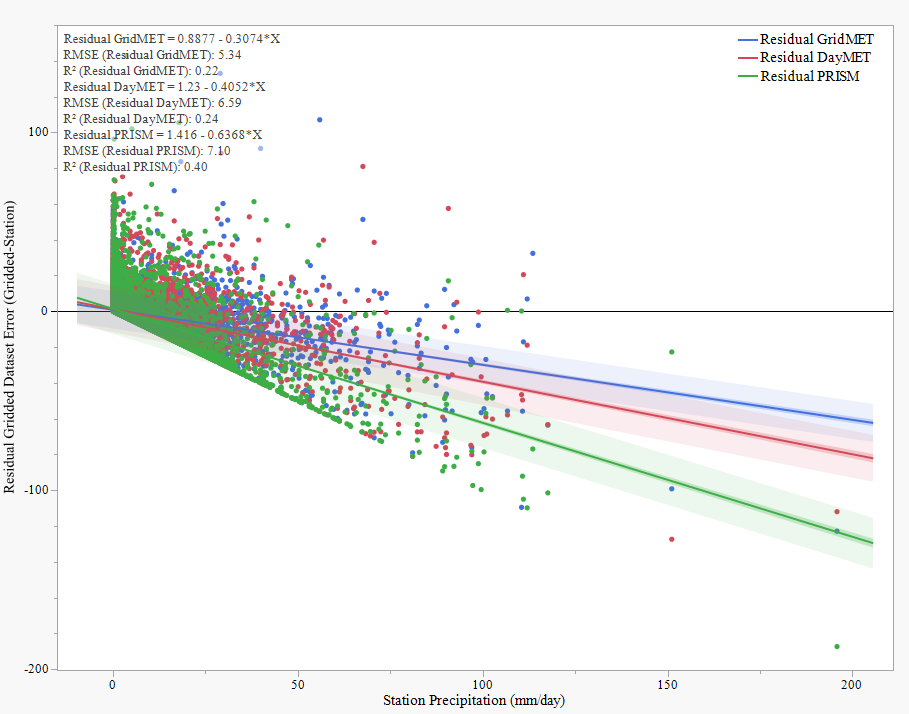


**B**

**A**

**Supplementary Figure 2.** Regressions between daily on-site weather station and three gridded weather datasets; GridMET, DayMET, and PRISM. Figure A indicates the regression between historical weather data sources, i.e., on-site weather station precipitation, i.e.,‘Station’ compared to the gridded products, GridMET, DayMET and PRISM. Figure B refers to the regression between the residual error value of each gridded data source where residual error=gridded-station precipitation data when compared to the on-site weather station precipitation (mm-month^-1^). For both figures, lines represent the regression and the surrounding like colors represent the confidence interval. Regressions were significantly different when *p*<0.05.
